# Efficient Small Extracellular Vesicles (EV) Isolation Method and Evaluation of EV-Associated DNA Role in Cell–Cell Communication in Cancer

**DOI:** 10.3390/cancers14092068

**Published:** 2022-04-20

**Authors:** Venkatesh Kumar Chetty, Jamal Ghanam, Srishti Anchan, Katarina Reinhardt, Alexandra Brenzel, Márton Gelléri, Christoph Cremer, Elena Grueso-Navarro, Markus Schneider, Nils von Neuhoff, Dirk Reinhardt, Jadwiga Jablonska, Irina Nazarenko, Basant Kumar Thakur

**Affiliations:** 1 Department of Pediatrics III, University Hospital Essen, 45147 Essen, Germany; venkateshkumar.chetty@uk-essen.de (V.K.C.); jamal.ghanam@uk-essen.de (J.G.); anchsana@gmail.com (S.A.); katarina.reinhardt@uk-essen.de (K.R.); markus.schneider@uk-essen.de (M.S.); nils.vonneuhoff@uk-essen.de (N.v.N.); dirk.reinhardt@uk-essen.de (D.R.); 2 Imaging Center Essen (IMCES), University Hospital Essen, 45147 Essen, Germany; alexandra.brenzel@uk-essen.de; 3 Institute of Molecular Biology (IMB), 55128 Mainz, Germany; m.gelleri@imb-mainz.de (M.G.); c.cremer@imb-mainz.de (C.C.); 4 Max Planck Institutes for Polymer Research and for Chemistry, 55128 Mainz, Germany; 5 Institute for Infection Prevention and Hospital Epidemiology, Medical Center-University of Freiburg, Faculty of Medicine, 79106 Freiburg, Germany; elenagru4@gmail.com (E.G.-N.); irina.nazarenko@uniklinik-freiburg.de (I.N.); 6 Department of Otorhinolaryngology, University Hospital Essen, 45147 Essen, Germany; jadwiga.jablonska@uk-essen.de; 7 German Cancer Consortium (DKTK), Partner Site Freiburg and German Cancer Research Center (DKFZ), 69120 Heidelberg, Germany

**Keywords:** small extracellular vesicles, pure EVs, EV isolation, EV characterization, exosomes, EV-DNA, cell-free DNA, extracellular dsDNA, EV communication, EV in cancer

## Abstract

**Simple Summary:**

Small extracellular vesicles (sEVs) released by all cell types function as a mediator in intercellular communication that can promote cell division and survival to remodel the tumor microenvironment to develop tumor invasion and metastasis. Even though dsDNA baggage is associated with all small EV populations, the functional role of EV-DNA in cancer remains poorly understood. This is due to a lack of methods allowing the efficient separation of small EVs (sEVs) from other non-sEV components. The main aim of our study was to develop an efficient sEV isolation method along with EV-associated DNA (EV-DNA) monitoring tool to evaluate the role of EV-DNA as a mediator of cell–cell communication in cancer. Our detailed small EV-DNA characterization confirmed that isolated sEVs using the TSU method (Tangential flow filtration + Size exclusion chromatography + Ultrafiltration) are free from contaminants such as cell-free and apoptotic bodies DNA, making TSU ideal for performing EV-DNA functional studies. Next, we revealed the exact EV-DNA distribution in the recipient cells using 3D image analysis and the association of EV-DNA with key cellular proteins, which may have an essential role in cancer. In the leukemia model, EV-DNA isolated from leukemia cell lines associated with mesenchymal stromal cells (MSCs), a crucial factor in the bone marrow (BM) microenvironment.

**Abstract:**

Small extracellular vesicles (sEVs) play essential roles in intercellular signaling both in normal and pathophysiological conditions. Comprehensive studies of dsDNA associated with sEVs are hampered by a lack of methods, allowing efficient separation of sEVs from free-circulating DNA and apoptotic bodies. In this work, using controlled culture conditions, we enriched the reproducible separation of sEVs from free-circulated components by combining tangential flow filtration, size-exclusion chromatography, and ultrafiltration (TSU). EV-enriched fractions (F2 and F3) obtained using TSU also contained more dsDNA derived from the host genome and mitochondria, predominantly localized inside the vesicles. Three-dimensional reconstruction of high-resolution imaging showed that the recipient cell membrane barrier restricts a portion of EV-DNA. Simultaneously, the remaining EV-DNA overcomes it and enters the cytoplasm and nucleus. In the cytoplasm, EV-DNA associates with dsDNA-inflammatory sensors (cGAS/STING) and endosomal proteins (Rab5/Rab7). Relevant to cancer, we found that EV-DNA isolated from leukemia cell lines communicates with mesenchymal stromal cells (MSCs), a critical component in the BM microenvironment. Furthermore, we illustrated the arrangement of sEVs and EV-DNA at a single vesicle level using super-resolution microscopy. Altogether, employing TSU isolation, we demonstrated EV-DNA distribution and a tool to evaluate the exact EV-DNA role of cell–cell communication in cancer.

## 1. Introduction

Extracellular Vesicles (EVs) are lipid bilayer bound vesicles released by all cell types into the extracellular environment, which differ in size, shedding mechanism, and function [1,2,3]. EVs contain unique biomolecular cargo, consisting of proteins, nucleic acids, and lipids [4,5,6,7,8]. In general, EVs can be classified into three types such as exosomes (30–150 nm), microvesicles (100–1000 nm), and apoptotic bodies (1–5 µm) [1,2,9,10]. Since markers are not well established based on EV origin, MISEV2018 guidelines urged EV researchers to use the term small EVs (sEVs) that are less than 200 nm in diameter and large EVs as the ones that are greater than 200 nm [3]. Small EVs have gained greater scientific research attention than large EVs in recent years. Many researchers have demonstrated that small EVs act as a functional mediator of intercellular communication in various instances, including cancer. In cancer, tumor cell-derived EVs regulate the tumor microenvironment through their cargo delivery promoting tumor progression, metastasis, and angiogenesis [8,11,12,13,14,15,16,17]. Although DNA has been associated with sEVs (EV-DNA), it is not extensively studied compared to other EV cargoes. Moreover, very little is known about the molecular mechanisms of EV-DNA packaging and their biological function in the recipient cells [1,5,7,13,18,19,20,21,22].

To demonstrate the presence of cancer biomarkers for liquid biopsy applications, it is not an issue to isolate heterogeneous EVs from blood samples and analyze them for cancer-specific mutations. However, it is very important for functional studies to purify and separate EV populations to discriminate the functional significance of DNA associated with a particular EV subtype [3]. Until now, most studies demonstrating the functional role of EV-DNA in the recipient cells have used classical ultracentrifugation (UC) or polymer-based techniques for EV isolation. However, these studies are still far from conclusive because these isolation methods are known to co-isolate other non-sEV populations, including cell-free DNA and apoptotic bodies, and also, cause morphological changes in the extracted vesicles [23,24,25,26]. Recently, Lazaro-Ibanez et al. attempted firstly to distinguish heterogeneous EV subpopulations based on DNA cargo and topology using UC-based iodixanol density gradient separation [27]. Although this method provides pure EVs for molecular characterization, multiple ultracentrifugation steps can result in sample loss affecting EV yield for downstream functional experiments [24]. 

Furthermore, not all laboratories have access to expensive ultracentrifugation devices; therefore, there is still the need to establish a method that can be performed in a laboratory bench setting yet provides sEVs that are qualified to perform both diagnostics and functional studies. To address these important problems, we have optimized two sEV isolation methods by combining tangential flow filtration (TFF) or PEG-based precipitation with conventional size-exclusion chromatography and ultrafiltration (SEC + UF) techniques, which we named TSU and PSU, respectively. The fractions were characterized according to the MISEV2018 guidelines [3]; the majority of small extracellular vesicles (sEVs) ranging from 30–200 nm were obtained in fraction 2 (F2) and fraction 3 (F3) of TSU and PSU. 

As we previously showed, we detected cancer-specific mutations in EV-DNA regardless of the isolation method used [5,21]. However, we found that only sEV fractions obtained from TSU entered the recipient cells and localized in various cell compartments, whereas sEVs isolated by PSU showed drastically reduced entry into recipient cells. In addition, we applied 3D imaging tools for the first time in the EV field to avoid artifacts obtained from 2D imaging and to accurately quantify the actual amount of EV-DNA entered inside the recipient cells. 

Previously, it was shown that EV uptake in the recipient cells occurs through clathrin-mediated endocytosis and micropinocytosis [28]. Moreover, it was recently demonstrated that the accumulation of cytoplasmic DNA arising from genomic DNA undergoing chromosomal duplication activates the cGAS-STING (cyclic GMP-AMP synthase-stimulator of interferon genes) cytosolic DNA-sensing pathway, which results in an innate immune response favoring the spread of cancer cells to distant organs [29]. Furthermore, T-cell-derived EVs carrying DNA can induce antiviral inflammatory responses via the cGAS/STING/IRF3 signaling cascade in priming dendritic cells [30]. Supporting these facts, we found that passenger EV-DNA associates with the endosome–lysosome compartment and cytoplasmic DNA sensors (cGAS/STING) in the recipient cells, which may have an important role in cancer. In addition, we found that EV-DNA derived from leukemia cell lines communicates with the critical component in the BM microenvironment, such as MSCs. Altogether, our work provides an efficient sEV isolation method and new tools to address EV-DNA-associated biology in cancer. 

## 2. Materials and Methods

### 2.1. Cells and Cell Culture

HeLa and HEK293T cells were cultured in DMEM high glucose media (Gibco, Paisley, UK) in a humidified incubator at 37 °C, 5% CO_2_ for 72 h. To perform acute myeloid leukemia (AML) diagnostic studies, MV4−11 and OCI−AML3 cell lines were cultured in RPMI 1640 media (Gibco, Paisley, UK). For EV functional studies, a HEK293T-CD63-GFP-transduced cell line received from Prof. Bernd Giebel (University Hospital Essen, Essen, Germany) was utilized and cultured like HEK293T cells. In this transduced cell line, CD63 is fused to the N-terminus of eGFP and was generated by co-transfection of HEK293T cells with the lentiviral plasmid pCL6-CD63-eGFP, the helper plasmid pCD/NL-BH, and the codon-optimized human foamy virus envelope encoding plasmid pcoPE01 [31]. 

Growth media was supplemented with 10% EVs-depleted fetal bovine serum (FBS; Gibco, Waltham, MA, USA) and 1% penicillin/streptomycin for all cell lines. EV-depleted FBS (FBS18) was obtained by ultracentrifugation at 100,000× *g* for 18 h utilizing a type 45 Ti fixed-angle titanium rotor (Beckmann Coulter, CA, USA). After 72 h, cell conditioned media (CCM) was collected and centrifuged at 500× *g* for 10 min followed by 3000× *g* for 20 min (4 °C) to remove cells, cell debris, and apoptotic bodies. All cell lines involved in this study were screened for mycoplasma contamination by performing PCR and authenticated using short tandem repeat (STR) DNA fingerprinting through IDEXX BioAnalytics, Germany. 

### 2.2. Isolation of Small EVs by TSU and PSU

To prepare TSU sEVs, CCM was first filtered using a 0.2 µm syringe filter to eliminate large EVs (>200 nm). Filtered CCM was concentrated up to 10 mL using tangential flow filtration (TFF-Easy; Hansa Biomed, Tallin, Estonia). Concentrated CCM was then loaded into a size-exclusion chromatography column (SEC) (qEV10; IZON Science, Christchurch, New Zealand) using 0.2 µm-filtered DPBS (Invitrogen, Waltham, MA, USA) as the running buffer. First, 20 mL was discarded as void volume, and right after, 5 mL fractions (F1–F5) were collected. The SEC fractions were further concentrated to 500 µL final volume using an Amicon Ultra-4 10 kDa centrifugal filter (Merck Millipore, Darmstadt, Germany). 

In order to prepare PSU sEVs, CCM was centrifuged at 6800× *g* for 45 min and then filtered using a 0.2 µm syringe filter. To the filtered CCM, precipitation agents such as 10% PEG6000 (Sigma, Darmstadt, Germany) along with 0.5% NaCl (Sigma, Darmstadt, Germany) were added and incubated overnight for 16 h at 4 °C. The next day, PEG-treated CCM was centrifuged at 1500× *g* for 30 min (40 °C) and the pellets were re-suspended in 2 mL of 0.2 µm-filtered DPBS [31]. Out of 2 mL of the PEG pellet, 100 µL (aliquot) was stored separately, and the volume was equalized again with filtered DPBS and loaded into the SEC column (qEV2; IZON Science, Christchurch, New Zealand). As mentioned before, 2 mL fractions (F1–F5) were collected and concentrated to 500 µL using an Amicon Ultra-2 10 kDa centrifugal filter (Merck Millipore, Darmstadt, Germany). In parallel, TSU and PSU sEVs were isolated from DMEM containing FBS18 (control without cells) by performing the same steps carried out with CCM.

### 2.3. Characterization of TSU and PSU sEVs

#### 2.3.1. Measurement of Particle Count and Total Protein Concentration

Firstly, the concentration of particles (particle count) present in various HEK293T and HeLa TSU and PSU sEV fractions (F1–F5) was determined by nanoparticle tracking analysis (NTA) using the ZetaView BASIC PMX-120 instrument (Particle Metrix GmbH, Inning am Ammersee, Germany) equipped with NTA 2.0 analysis software. Using 100 nm standard beads, the instrument was calibrated, and the following settings were used: positions—11, cycles—5, minimum size—5 nm, maximum size—150 nm, trace length—15 s, sensitivity—75%, shutter speed—75 ms and frame rate—30. Diluted TSU and PSU sEV fractions (100- to 1000-fold) were loaded into the NTA instrument and the values were recorded. Next, micro-BCA assay (Thermo Scientific, Waltham, MA, USA) was performed following the manufacturer’s protocol to determine the free protein concentration of various sEV fractions. Then, EV purity was determined by the ratio of particle count determined by NTA and free protein concentration determined by micro-BCA. 

#### 2.3.2. Transmission Electron Microscopy (TEM)

Negative staining was performed at the Electron Microscopy Unit (EMU) of the Imaging Center Essen (IMCES) for HEK293T and HeLa TSU and PSU sEV fractions that have more purity (F2 and F3) along with F4 that has less purity. In addition, FBS18 sEV fractions were included as a negative control. Briefly, 3 µL of sEV fractions (F2–F4) was added onto a Formvar- and carbon-coated 200 mesh copper grid (#S162, PLANO GmbH, Wetzlar, Germany) which had a hydrophilic surface due to being exposed to glow discharging for 30 s at 15 mA (easiGlow™, TedPella Inc., Redding, CA, USA). Samples were then negatively stained by placing the grid on the top of the droplet of 1.5% aqueous Phosphotungstic acid solution (*w*/*v*, 2635.1, Carl Roth, Karlsruhe, Germany) for a minute. Excess liquid was removed using filter paper, and the grids were allowed to dry for at least five minutes under ambient air. Images were acquired using JEOL JEM 1400Plus (JEOL Ltd., Tokyo, Japan) operating at 120 kV and with a 4096 × 4096 pixels CMOS camera (TVIPS, Gauting, Germany). Image acquisition software EMMENU (version 4.09.83) was used for taking 16-bit images. Image post-processing and analysis were carried out using ImageJ (version 1.52a) to determine the average diameter of sEVs. 

#### 2.3.3. Western Blot Analysis

Equal amounts (100 µL) of HEK293T, HeLa, and FBS18 TSU and PSU sEVs (F1–F5) were concentrated to 10–12 µL using an Amicon Ultra-0.5 10 kDa centrifugal filter tube (Merck Millipore, Darmstadt, Germany). Both whole-cell lysate (30 µg; prepared using RIPA buffer: ThermoFisher Scientific, Waltham, MA, USA) and EV fractions (F1–F5) were solubilized using 4X Laemmli buffer (Biorad, Feldkirchen, Germany) in reducing conditions at 95 °C for 10 min. Samples were then separated on a NuPAGE 4–12% gel, Bis-Tris, 1.0 mm, (ThermoFisher Scientific, Waltham, MA, USA) along with a pre-stained protein ladder at 100 V for 2 h. Transfer of resolved proteins onto a 0.4 µm PVDF membrane (Merck Millipore, Darmstadt, Germany) was performed following standard semidry conditions. Membranes were blocked in 5% milk blocking solution (Carl Roth, Karlsruhe, Germany) in 1X TBST buffer containing 0.1% Tween20 for 60 min. Membranes were incubated with primary antibodies diluted in 1X TBST containing 0.05% Tween20 (1:1000) overnight at 4 °C [Rabbit-α-TSG101 (Sigma, Cat. No: HPA006161); Rabbit-α-Calnexin (Abcam, Cat. No: ab22595); Mouse-α-CD81 (Biolegend, Cat. No: 349502); Rabbit-α-Hsp70 (System Biosciences, Cat. No: EXOAB-Hsp70A-1); Rabbit-α-Synthenin (Abcam, Cat No: ab133267).

Unbound antibodies were removed by washing the membranes 4X with 1X TBST and then incubated with the corresponding HRP-conjugated secondary antibody (1:10,000 dilution) at RT for 90 min (anti-mouse IgG, HRP-linked: Cell Signaling Technology, Danvers, MA, USA, Cat. No: 7076; anti-rabbit IgG, HRP-linked: Cell Signaling Technology, Danvers, MA, USA, Cat. No: 7074; goat anti-rabbit for detection of Hsp70: System Biosciences, Cat. No: EXOAB-Hsp70A-1). Proteins were visualized using ECL Prime Western Blotting Detection reagents (ThermoFisher Scientific, Waltham, MA, USA) by scanning the membrane on a Fusion FX machine (Vilber Lourmat GmbH, Eberhardzell, Germany).

#### 2.3.4. Bead-Assisted Flow Cytometry

HEK293T and HeLa TSU and PSU sEV fractions (F1–F5) were analyzed by flow cytometry for semi-quantitative detection of classical EV proteins (CD9 and CD63), apoptotic bodies (phosphatidylserine (PS)), and apolipoprotein (Apo-B). To detect the PS level present in sEVs, annexin-V antibody was employed. A volume of 20 µL of sEV fractions was incubated with 5 µL of aldehyde-sulfate latex beads (4 µM; Invitrogen) for 30 min at RT in a rotating platform shaker. Then 200 µL of 0.1 µm-filtered 1X DPBS was added and incubated for another 30 min at RT. Afterwards, 300 µL of DPBS was added and centrifugation was performed at 2000× *g* for 5 min to remove the unbound beads in a way that 50 µL of the beads–EV complex was left. To this, 20 µL of a 5% BSA solution in DPBS was added for blocking and incubated for 30 min at RT. DPBS washing and centrifugation were performed as mentioned before. In the last step, staining was carried out using 5 µL of antibody conjugated with different fluorochrome for 30 min at RT (antihuman CD9-PE (MEM61, Exbio, Cat. No: 1P-208-T100); antihuman CD63-APC (Mem259, Exbio, Cat. No: 1A-343-T100); antihuman Apo-B-FITC (polyclonal, Abcam, Cat. No: ab27637); annexin-V-FITC (N/A, BD Biosciences, Cat. No: 560931). A final wash step was performed to remove the unbound antibodies. In addition, various controls were included in the analysis. Beads only and beads with antibody controls were used to select the single beads (monomer) and positive population. Beads with BSA and beads with BSA+ antibody controls were included in determining if BSA bound with any antibodies used. Data were acquired in conventional flow cytometers (BD FACS Aria, BD Biosciences, Heidelberg, Germany, and MACS Quant, Miltenyi Biotech, Bergisch Gladbach, Germany) and analyzed using FlowJo software V10 to determine the mean fluorescence intensity (MFI). MFI relative to the negative control in TSU and PSU sEV fractions was evaluated and depicted on the y-axis. EV purity was also determined based on the values of EV tetraspanins obtained from this method and the free protein concentration was determined by micro-BCA assay. 

### 2.4. Application of TSU and PSU sEVs for Diagnostics and Functional Studies

#### 2.4.1. MV4−11 and OCI−AML3 sEVs for AML Diagnostics

TSU and PSU sEV fractions (F2–F4) obtained from MV4−11 and OCI−AML3 CCM were characterized by performing Western blot with CD81, TSG101, and calnexin. EV-DNA was isolated from these fractions using the QIAamp DNA Micro Kit (#56304, Qiagen) according to the manufacturer’s instructions, and the concentration was determined using a Nanodrop 1000 spectrophotometer (ThermoFisher Scientific, Waltham, MA, USA). As shown before, GeneScan-based fragment-length analysis was performed to determine if MV4−11 and OCI−AML3 TSU and PSU sEVs (F2–F4) qualify to detect AML-specific mutations (FLT3 and NPM1 mutations) [21]. 

#### 2.4.2. Incubation of HEK-CD63-GFP sEVs with HeLa Cells

Around 25,000 HeLa cells were seeded on a 24-well plate containing sterile 12 mm microscopic coverslips (ThermoFisher Scientific, Waltham, MA, USA). After 24 h, normal growth media was replaced with FBS18 media. Thirty microliters of HEK-CD63-GFP TSU and PSU sEV fractions (F1–F5) was added to the corresponding wells and incubated at 37 °C for 48 h. After 48 h, cells were washed once with PBS and 0.01% PBST before being fixed using 4% PFA (Alfa Aesar, Kandel, Germany) for 15 min at RT. Then, 3% BSA in PBS was added and removed, followed by 2X PBS washes. Permeabilization was performed using 0.5% Triton^®^-X100 (Merck Millipore, Darmstadt, Germany) in PBS for 20 min at RT, after which cells were washed with 3% BSA and PBS as mentioned before. Nucleus staining was performed using DAPI solution (0.2 µg/mL, Biolegend) in PBS for 10 min at RT in the dark, followed by 2X PBS washes. Coverslips were mounted carefully using a few drops of Fluoromount (Southern Biotech, Birmingham, AB, USA) on microscopic glass slides. Images were acquired using a confocal microscope (Leica TCS SP8, Wetzlar, Germany), and the mean GFP fluorescence intensity was measured using ImageJ 1.52a (NIH, Bethesda, MD, USA). 

#### 2.4.3. Zeta Potential

Next, we wanted to investigate whether the biophysical property of EVs, such as zeta potential, plays a crucial role in mediating its successful transfer into recipient cells. Using the ZetaView BASIC PMX-120 instrument, the zeta potential of HEK-CD63-GFP TSU and PSU EVs (F1–F5) was measured at 25 °C under the following settings: max size: 200, min size: 5, min brightness: 20. 

### 2.5. Transfer of Foreign EV-DNA in the Recipient Cells

#### 2.5.1. Labelling of EV-DNA with EdU

In order to facilitate EV-DNA-based functional studies, 5 µM of 5-ethynyl-2′-deoxyuridine (EdU; ThermoFisher Scientific) solution was added to HEK-CD63-GFP cells after a few hours of seeding (i.e., when cells had attached to the dishes). EdU is a thymidine analog incorporated into newly synthesized DNA during active DNA synthesis; thereby, cells treated with EdU release EVs in which DNA is labeled with EdU. sEVs were isolated from HEK-CD63-GFP cells treated with EdU using TSU and PSU.

#### 2.5.2. Incubation of sEVs Containing EV-DNA-EdU with HeLa Cells

Thirty microliters of the HEK-CD63-GFP TSU and PSU sEVs (F1–F5) containing EV-DNA-EdU was incubated with HeLa cells seeded on a 24-well plate containing sterile 12 mm microscopic coverslips at 37 °C for 48 h. In parallel, MV4−11 sEVs containing EdU were added to mouse mesenchymal stromal cells (OP9). Fixation and permeabilization were performed as explained before. If cell membrane staining was desired, cells were treated with wheat germ agglutinin (WGA) Alexa Fluor™ 488 conjugate (W11261, Invitrogen) for 10 min well after fixation, before permeabilization. An EdU click-it reaction was carried out using the Click-iT™ EdU Alexa Fluor 647 Imaging Kit (C10640, ThermoFisher Scientific) following the manufacturer’s instructions. Images were acquired by confocal microscopy in the corresponding channels (blue—DAPI, green—CD63+ EVs, and red—EdU). Using ImageJ, the red fluorescence signal corresponding to EV-DNA was quantified. 

#### 2.5.3. Three-Dimensional (3D) Image Analysis

For 3D analysis, the Leica SP8 confocal system with a 63X objective was used. Z-stack imaging of the cells was performed with Nyquist sampling for subsequent deconvolution of the data set with Huygens. After that, the data were loaded into Imaris for 3D analysis to obtain the exact amount of EV-DNA present inside the cell, inside the nucleus, and those close to the cell membrane, by using the Imaris surface function to distinguish between the cell compartments and the spot function to count the EV-DNA.

### 2.6. Single EV Imaging

For single-molecule localization measurements, a custom-built inverted microscope equipped with 488 nm, 561 nm, and 640 nm lasers was used [32]. The laser beams were combined by dichroic mirrors and then expanded and focused on the objective’s back focal plane (HCX PL APO 100×/NA 1.47 OIL, Leica). Fluorescence light was separated from excitation light using a dichroic mirror (Chroma, zt405/488/561/647rpc). A tube lens focused the fluorescence light onto an sCMOS camera (PCO edge 4.2, PCO), resulting in an effective pixel size of 65 nm. A cylindrical lens was inserted into the detection path to introduce astigmatism for 3D localization measurements. The detection path was additionally equipped with emission filters (HC520/35 Semrock, ET600/50 Chroma, ET655 Chroma) to reduce bleed-through from different fluorescence channels. 

HEK-CD63-GFP TSU EVs (F2 and F3) containing EdU were embedded in Vectashield H-1000 mounting medium (Vector Laboratories, Burlingame, CA, USA) on the day of measurement. CD63-GFP and Edu-Alexa Fluor 647 were excited using 488 nm and 640 nm lasers and imaged with emission filters HC520/35 (GFP) and ET655laser (Alexa647), respectively. Excitation powers were set to 0.8 kW/cm2 to 3 kW/cm^2^ for both lasers. A stack of 5000 images with an exposure time of 50 ms was acquired for each channel. For data analysis, reconstruction of SMLM data was performed with the ThunderSTORM plugin [33] in Fiji [34]. A detection threshold of one standard deviation of the applied wavelet filter was used during the detection process. Fluorescence signals visible in consecutive frames within a radius of 20 nm were merged into one detection event to avoid over-counting of molecules. Super-resolution images were then generated as 2D histograms with a pixel size of 20 nm.

### 2.7. Detailed Characterization of HEK293T TSU EV-DNA

#### 2.7.1. Isolation of EV-DNA before and after dsDNAse Treatment

To determine whether DNA is associated with the outer membrane or inside EVs, we pre-treated 100 µL of HEK293T TSU sEVs (F1–F5) with 6 µL of dsDNAse (ThermoFisher Scientific, Waltham, MA, USA) along with 10X dsDNAse buffer at 37 °C for 5 min and the reaction was stopped by adding 0.5 M EDTA. Then, EV-DNA was extracted from both intact HEK293T TSU sEVs and sEVs pre-treated with dsDNAse (F1–F5) using the QIAamp DNA Micro Kit. Three microliters of these extracted EV-DNA samples was again treated with dsDNAse in the same manner to evaluate if single-stranded DNA is present inside sEVs. Finally, 5 µL of HEK293T EV-DNA samples (four sets) and genomic DNA was loaded on a 2% agarose gel and run at 100 V for one hour. After one hour, the agarose gel was stained with SYBR Gold nucleic acid dye (ThermoFisher Scientific, Waltham, MA, USA) for 30 min and the gel image was acquired. 

#### 2.7.2. Next-Generation Sequencing (NGS) 

HEK293T TSU sEVs (F1–F5) and HEK293T gDNA samples were divided into two: one was pre-treated with dsDNAse to digest extra vesicular dsDNA and the other one remained untreated prior to DNA extraction and NGS. DNA was extracted in the same way as mentioned before. After DNA extraction, samples were processed for NGS using the NEB Next Ultra II FS DNA Library Prep Kit for Illumina (New England BioLabs, Frankfurt, Germany) following the manufacturer protocols of inputs ≤100 ng for EV-DNA samples; and large fragment sizes (>550 bp) for gDNA. DNA library quality was assessed on an Agilent 2200 TapeStation system using High Sensitivity D1000 ScreenTape (Agilent Technologies, Santa Clara, CA, USA). Once the expected band size based on fragmentation time was confirmed for all the samples, sequencing was performed on an Illumina MiSeqDX Sequencer in research mode with 156 bp paired-end reads using the MiSeq Reagent Kit v2 (300-cycle). After obtaining data, sequencing adapters were removed by Trimmomatic software [35], adapter-free read pairs were mapped to the hg38 genome, and read counts per chromosome were accessed by samtools idxstats [36]. 

### 2.8. Immunofluorescence

HeLa cells seeded on a 24-well plate were incubated with 30 µL of HEK293T TSU EVs with EdU (F2 and F3) at 37 °C for 48 h. Fixation, permeabilization, and click-it EdU staining were performed as previously explained. Then, blocking was performed using 1% BSA in PBS for 30 min at RT followed by incubation with primary antibody: rabbit-α-Rab5 (1:250, Abcam, Cambridge, UK); Rab7 (1:1000, Abcam, Cambridge, UK); Lamin (1:1000, Abcam, Cambridge, UK); cGAS (1:500, Cell signaling technology, Danvers, MA, USA); STING (1:1000, Abcam, Cambridge, UK) at RT for 70 min. Subsequently, 3X PBS washes were performed and then incubated with anti-rabbit Alexa488 (1:500, Abcam, Cambridge, UK) for 45 min at RT. Following 3X PBS washes and DAPI staining, images were obtained by confocal microscopy. 

### 2.9. Statistical Analysis

Statistical analyses were performed using GraphPad Prism 7.0. All data sets were represented as the mean ± S.E.M (shown with error bars) obtained from three independent experiments. One unpaired *t*-test per row was performed to assess the statistical significance, and *p* values were determined using the Holm–Sidak method. *p*-values < 0.05 were considered to be significant. Data that were found to be statistically significant were represented in the graphs as: * for *p* < 0.0332, ** for *p* < 0.021, *** for *p* < 0.0002, and **** for *p* < 0.0001. 

## 3. Results

### 3.1. Isolation and Characterization of HEK293T TSU and PSU sEVs 

**Figure 1:**
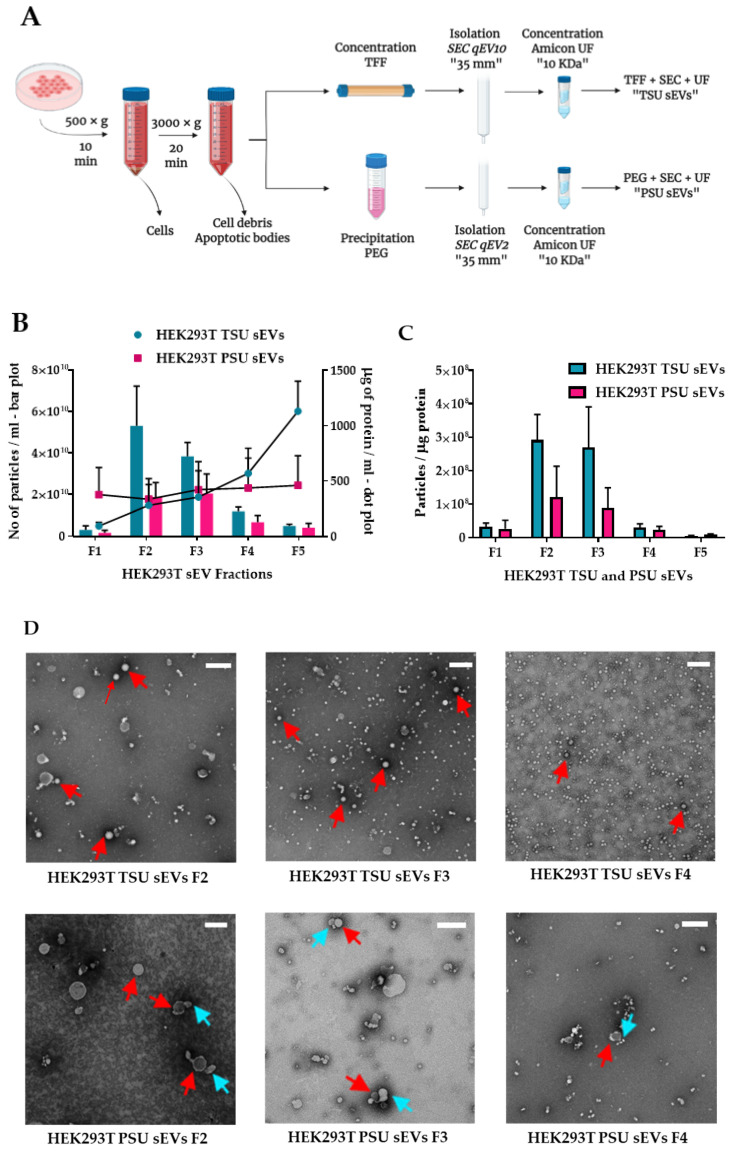

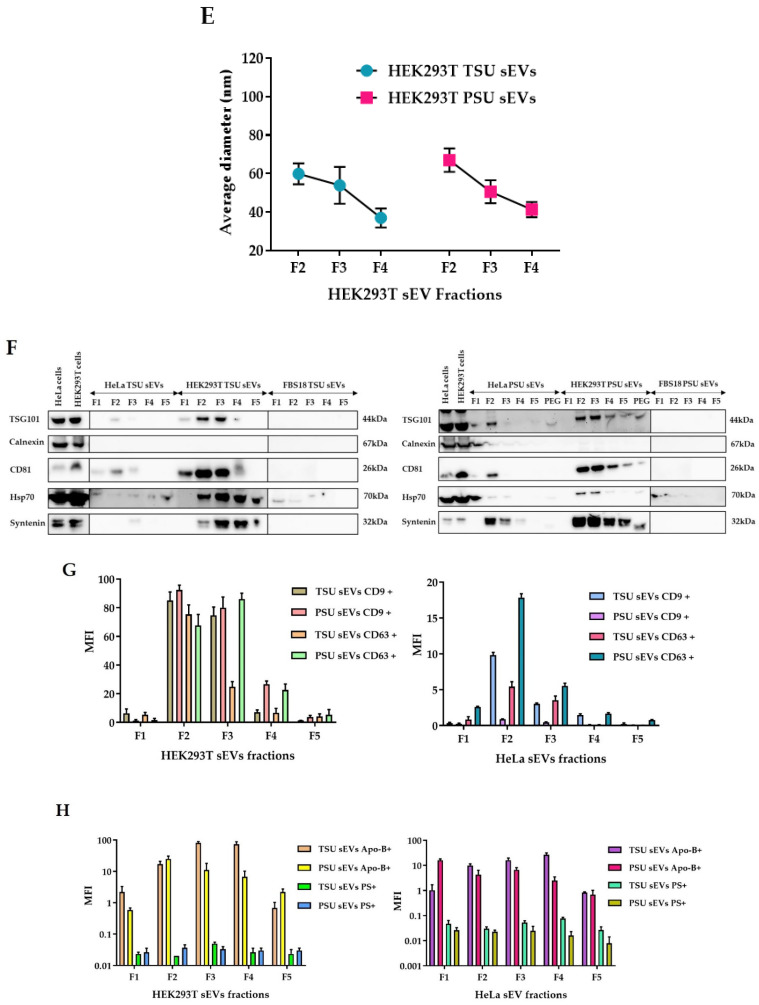
Isolation and characterization of TSU and PSU sEVs. (**A**) Schematic representation of the steps involved in the isolation of sEVs from CCM using TSU and PSU. (**B**) Mean values of particle count and free protein concentration (*n* = 3) in HEK293T TSU and PSU sEV fractions determined by NTA (left y-axis, bar plot) and micro-BCA (right y-axis, dot plot). (**C**) Measurement of EV purity from the ratio of particle count to free protein concentration. Data correspond to the mean ± S.E.M of different HEK293T TSU and PSU sEV fractions purity values from three independent experiments. (**D**) Imaging of TEM negative staining of HEK293T TSU and PSU sEVs (F2–F4) (6000× magnification). Red arrows indicate sEVs and aberrant bubbles found in PSU sEVs are shown by blue arrows. Scale bars: 200 nm. (**E**) Evaluation of average size diameter of HEK293T sEVs present in TSU and PSU sEV fractions (F2–F4) utilizing TEM negative staining images(*n* = 3) using ImageJ. (**F**) Western blot analysis of TSU and PSU sEVs with various EV canonical markers and calnexin. Whole-cell extract (positive control), HeLa, and HEK293T sEV fractions (F1–F5) including FBS18 sEV fractions (negative control) were used. Each black outer line box indicates the samples that were run together. Semi-quantitation of (**G**) EV tetraspanin markers (CD9 and CD63) and (**H**) other non-small EVs contaminants such as apolipoprotein-B [Apo-B] and PS^+^ apoptotic bodies in TSU and PSU sEV fractions using bead-based flow cytometry. Error bars represent the mean ± S.E.M from three independent experiments.

Enrichment, fractionation, and isolation of homogenous vesicular and non-vesicular particles were established from HEK293T and HeLa CCM using TSU and PSU methods as explained in Figure 1A. To characterize sEVs as explained in Webber and Clayton, 2013, [37], particle count was determined in each fraction using NTA, and total protein concentration was determined using micro-BCA. We observed that the highest purity of sEV preparations determined by the highest particle number (30–200 nm) and lowest protein content was obtained in both TSU and PSU isolation methods (Figure 1B,C and Supplementary Figure S1A,B). For qualitative characterization, we selected fractions, F2 and F3, with the highest EV purity for negative staining, imaged using TEM, and compared to fraction F4 as the one with the least EV purity, according to our criteria. Indeed, particles present in F2 and F3 possessed an sEVs morphology with an intact membrane with a size ranging from ~30–200 nm in diameter (indicated by red arrows) irrespective of the cell lines or isolation methods used. (Figure 1D and Supplementary Figure S1C). However, some aberrant bubbles were found on the outer surface of PSU sEVs (indicated by blue arrows). Using TEM images, the average size of particles found in fractions (F2–F4) was calculated using ImageJ and is shown in Figure 1E and Supplementary Figure S1D. 

Qualitative analysis of TSU and PSU sEV fractions was performed by Western blot analysis with various canonical EV markers (TSG101, CD81, Hsp70, and syntenin). The negative signal of calnexin (an integral endoplasmic reticulum protein) further confirmed the isolation of sEVs. Supporting the TEM data, we found that all canonical EV proteins were enriched in both HEK293T TSU and PSU sEVs (F2, F3). However, the level of canonical protein markers found in HeLa TSU and PSU sEVs (F2, F3) is less compared to HEK293T sEVs. A weak band of calnexin was observed in some PSU sEV fractions. As previously shown [38], we also observed heat shock protein 70 (Hsp70) band in some TSU and PSU sEV fractions isolated from FBS18 media alone (control without cells), thereby suggesting that a small proportion of Hsp70+ sEVs are derived from FBS growth supplement used in the cell culture media (Figure 1F and Supplementary Figure S1E).

Next, we performed bead-assisted flow cytometry for further qualitative characterization of sEV fractions since this method is sensitive and time saving. To do so, we determined the level of surface-EV markers (CD9 and CD63 tetraspanins) and non-small EV contaminants such as phosphatidylserine (PS^+^) apoptotic bodies and apolipoprotein-B (Apo-B) in all sEV fractions [39]. Positive populations were selected as shown in Supplementary Figure S1F,G. As expected, CD9 and CD63 are primarily present in F2 and F3 of TSU and PSU sEVs from HEK293T cells. In line with the WB results, we observed that CD9 and CD63 signals are weaker in HeLa sEVs irrespective of the isolation method used (Figure 1G). EV purity was also calculated based on the EV tetraspanin value obtained from flow cytometry and free protein concentration determined by micro-BCA. Indeed, we determined that fractions F2 and F3 have more EV purity than other fractions (Supplementary Figure S1H). Interestingly, we found that the level of PS^+^ apoptotic bodies is close to zero in all TSU and PSU sEVs (F1–F5), indicating their absence. On the other hand, Apo-B contamination was lower in PSU sEVs than in TSU sEVs (Figure 1H). Overall, our results indicate that both TSU and PSU methods are able to isolate sEVs with high purity.

### 3.2. Assessment of TSU and PSU sEVs for Diagnostic and Functional Studies

**Figure 2:**
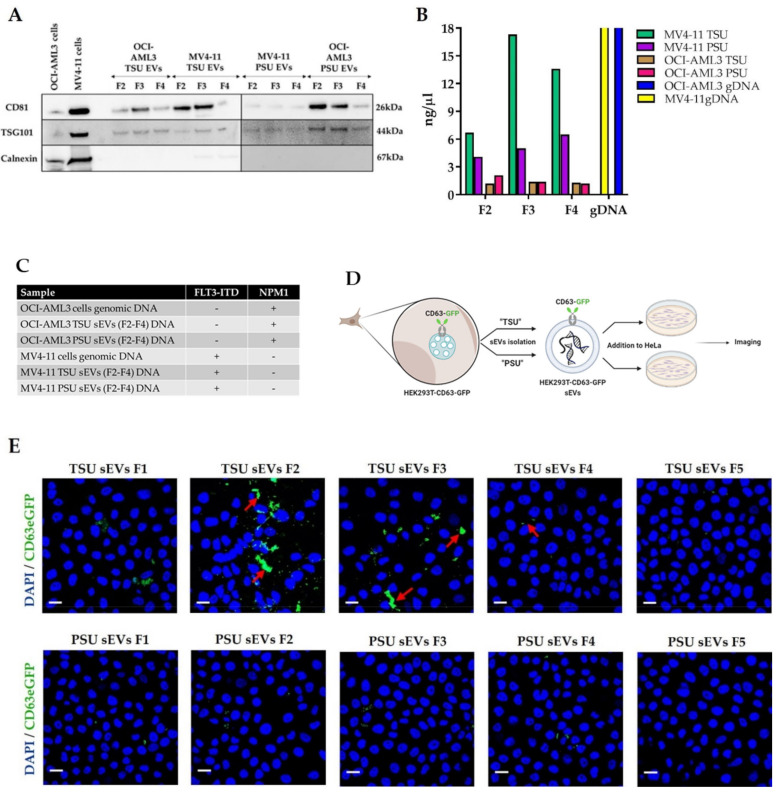

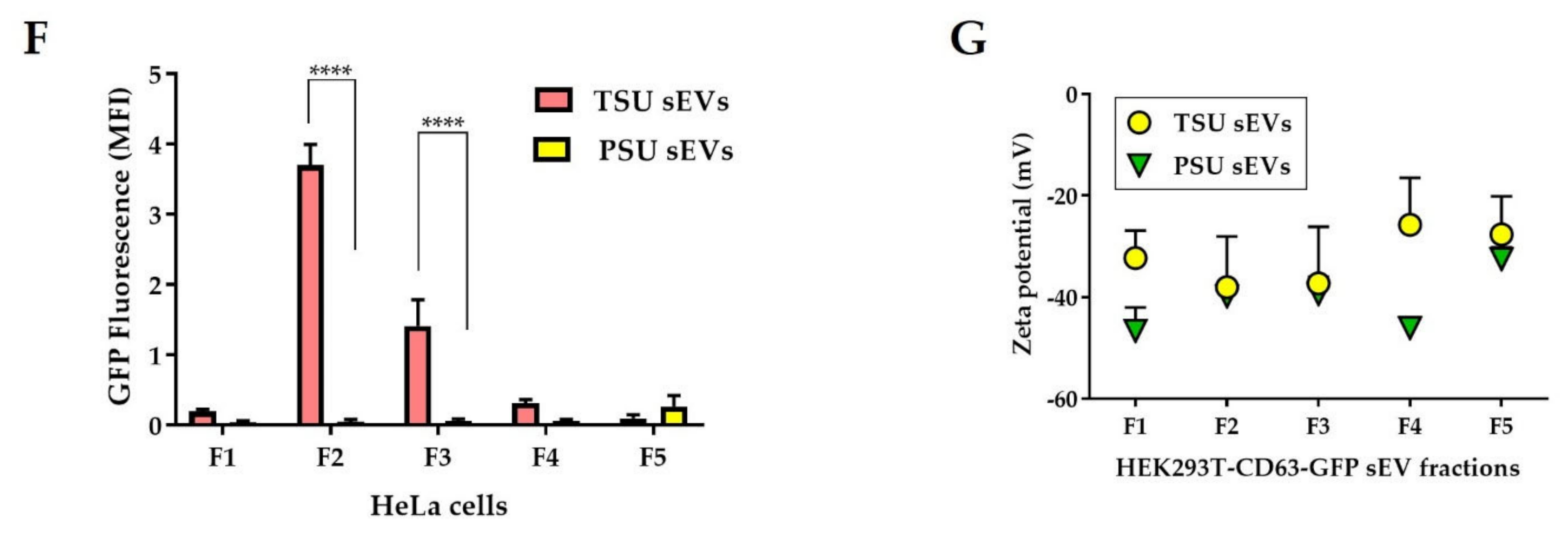
Utilization of sEVs for diagnostic and functional studies. (**A**) Immunoblot analysis of TSU and PSU sEV fractions (F2–F4) obtained from OCI−AML3 and MV4−11 with CD81, TSG101, and calnexin. (**B**) Comparison of EV-DNA concentration of OCI−AML3 and MV4−11 TSU and PSU sEV fractions (F2–F4). (**C**) GeneScan-based fragment-length analyses for detecting FLT3−ITD and NPM1 mutations both in genomic DNA and in their corresponding TSU and PSU sEVs. (**D**) Schematic diagram showing how sEVs obtained from HEK-CD63-GFP stable cell line can be helpful for EV functional studies. (**E**) Immunofluorescence staining of HeLa cells cultured with HEK293T-CD63-GFP TSU and PSU sEVs. Representative confocal images showing cell nuclei stained by DAPI (blue) and GFP fluorescence (green) indicating CD63^+^ sEVs (shown by red arrows). Scale bar: 10 µm. (**F**) Quantitative analysis of GFP fluorescence derived from CD63^+^ TSU and PSU sEVs (*n* = 3). (**G**) Zeta potential of HEK-CD63-GFP TSU and PSU sEVs (*n* = 3) measured using NTA. Data are shown as the mean ± S.E.M. **** *p* < 0.0001.

Previously, we [5] and others [7] have shown that tumor-derived exosomes, now known as small EVs, carry double-stranded DNA (dsDNA) that mirrors the mutational status of the donor cancer cells. Nevertheless, whether the EV-DNA derives from apoptotic bodies or dead cells is currently debated. Since TSU and PSU sEVs exhibited low apoptotic markers, we first evaluated if they qualify to perform diagnostic studies in the clinical setting. For this, we selected F2, F3, and F4 of TSU and PSU sEVs derived from AML cell lines (OCI−AML3 cell line bearing NPM1 mutation and MV4−11 cell line bearing FLT3−ITD mutation) to perform mutational analysis as only these fractions revealed the presence of sEVs (Figure 2A). EV-DNA was isolated (Figure 2B) and subjected to GeneScan-based fragment-length analysis to detect AML-specific mutations (FLT3−ITD and NPM1 mutations). The results showed that NPM1 and FLT3−ITD mutations were successfully detected in TSU and PSU sEVs (F2–F4) (Figure 2C). These data are consistent with our previous studies, thereby proving that both TSU and PSU sEVs (F2–F4) qualify to perform diagnostic studies [8].

To evaluate if isolated sEVs enable us to perform EV-based functional studies, we compared the uptake of TSU and PSU sEVs by HeLa cells. For this, a HEK293T-CD63-GFP transfected cell line was used that allowed us to track CD63^+^ EVs through a GFP signal (Figure 2D). Interestingly, we found TSU, but only to a limited extent F2 and F3 PSU sEVs were taken up by HeLa cells as indicated by the GFP signal detected in the cells by confocal microscopy (Figure 2E). For the quantitative analysis, the level of GFP fluorescence derived from CD63^+^ TSU and PSU sEVs on HeLa cells was determined using ImageJ and illustrated in Figure 2F. Studies have been focused on the influence of zeta potential in cellular uptake and EV stability, which is the measurement of surface potential that determines the magnitude of the electrical double layer repulsion [40,41]. In this respect, we investigated the zeta potential differences between TSU and PSU sEVs that could explain different up-take rates. No difference in zeta potential was found on both F2 and F3 of TSU and PSU sEVs (Figure 2G). Alternatively, we hypothesized the possibility that the PEG chemical present in PSU sEVs somehow inhibits the entry of sEVs into recipient cells. 

### 3.3. Localization of EV-DNA in the Cell Membrane, Cytoplasm and Nucleus

**Figure 3:**
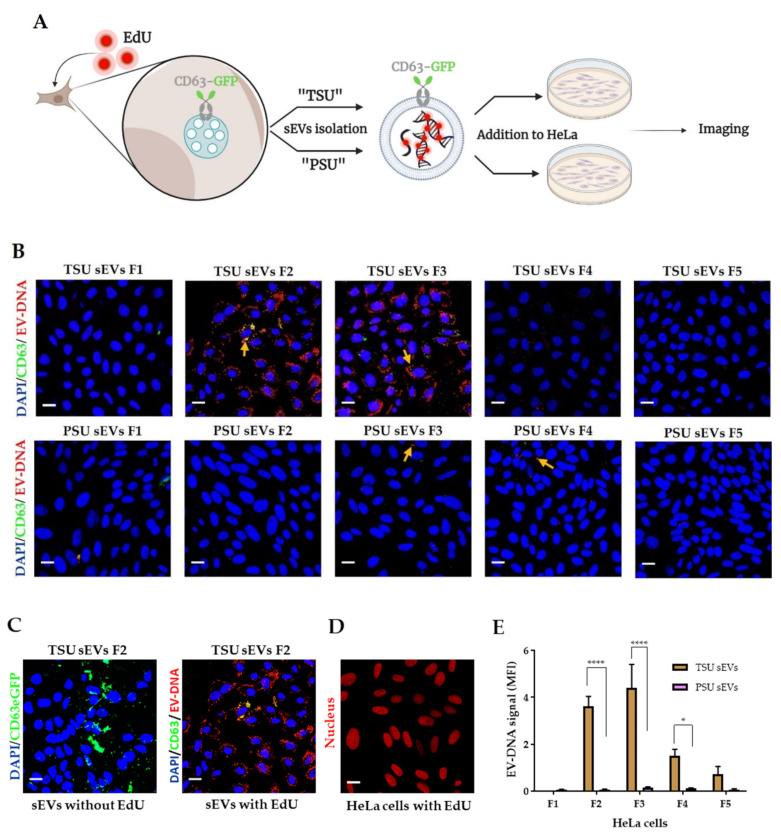

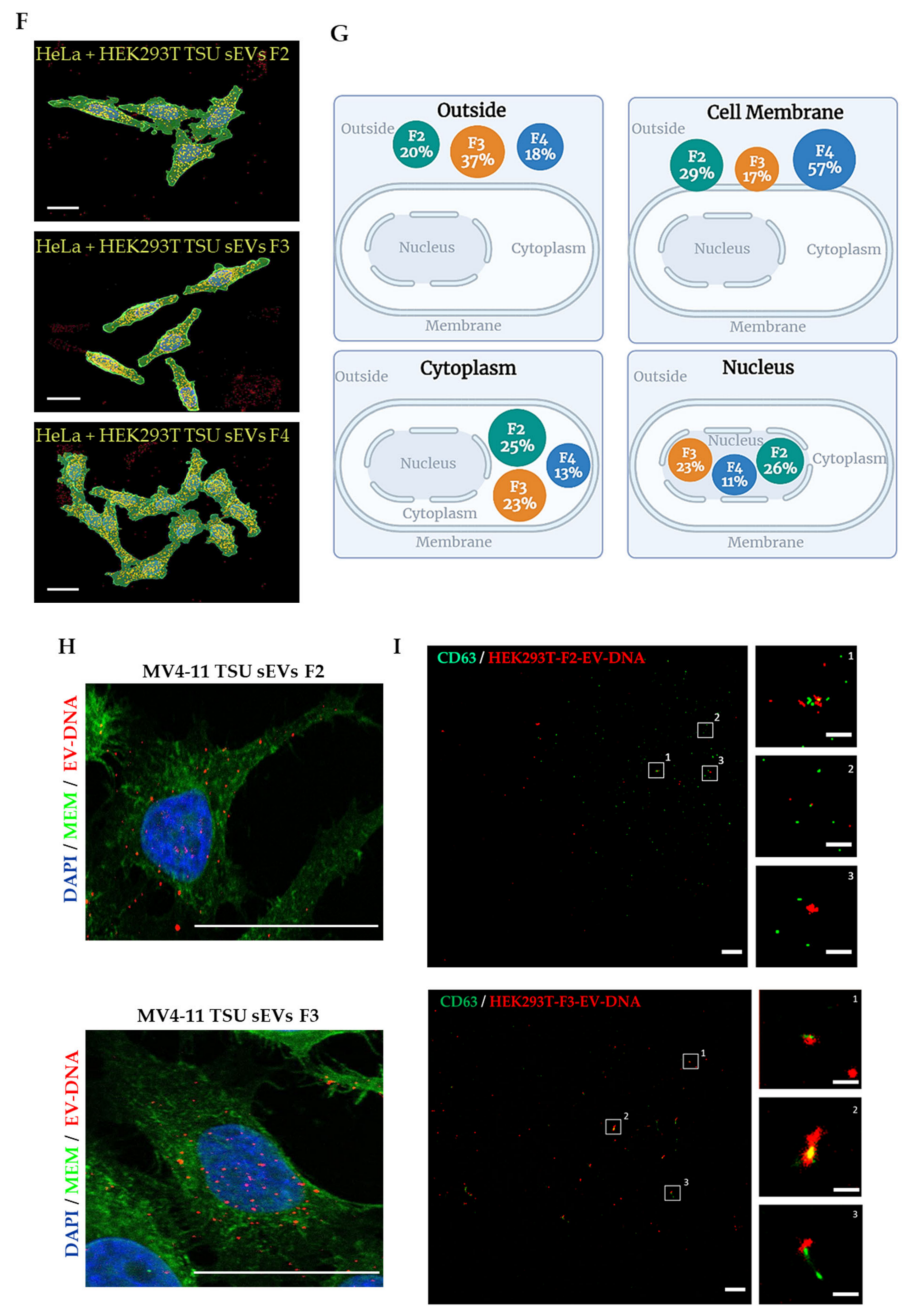
EV-DNA uptake in the recipient cells. (**A**) Figure explaining the incorporation of EdU into cellular DNA, thereby labeling EV-DNA with EdU for functional studies. (**B**) Immunofluorescence staining of HeLa cells transferred with HEK293T-CD63-GFP sEVs (F1–F5) containing EV-DNA-EdU. Representative confocal images illustrating cell nuclei (blue) stained by DAPI, GFP (green) derived from transferred CD63+ EVs, and localized foreign EV-DNA inside HeLa cells (red) detected using click-it azide-Alexa647. Yellow arrows show the co-localization of CD63^+^ EVs (GFP) and transferred EV-DNA (red). (**C**) Quenching of GFP fluorescence due to copper ions derived from click-it EdU staining kit. (**D**) HeLa cells after EdU treatment—positive staining control. Scale bar: 10 µm. (**E**) Quantification of EdU signal to determine EV-DNA transfer efficiency (*n* = 3). (**F**) Three-dimensional image analysis for the accurate quantification of EV-DNA. Representative images obtained from IMARIS showing HeLa cells educated with HEK293T TSU sEVs. Scale bar: 20 µm. (**G**) Measurement of foreign EV-DNA found outside recipient cells and at the cell membrane, cytoplasm, and nucleus. (**H**) Imaging of OP9 cells incubated with MV4−11 sEVs containing EV-DNA-EdU. Representative confocal images showing nuclei staining by DAPI (blue), cell membrane staining by WGA (green), and EV-DNA-EdU staining by click-it kit (red). (**I**) Super-resolution microscopy imaging of HEK293T-CD63-GFP TSU sEVs (F2 and F3) containing EdU. CD63^+^ EVs-GFP is shown in green, and EV-DNA-Alexa647 is shown in red. Scale bar: 2 µm and Scale bar insets: 0.5 µm. Data are shown as the mean ± S.E.M. * *p* < 0.0332 and **** *p* < 0.0001.

Horizontal transfer of DNA fragments via EVs from tumor to healthy stromal cells can be a detrimental factor in cell-to-cell communication in cancer. Therefore, we aimed to determine the capability of different sEVs fractions (F1–F5) to transport DNA into recipient cells. For this, the HEK293T-CD63-GFP cell line was incubated with 5-ethynyl-2′-deoxyuridine (EdU) for 72 h to label DNA. As a result, EVs containing DNA were stained with EdU. Later, both genomic DNA and sEVs were isolated from these EdU-treated cells. EdU was detected based on a click-it reaction on HeLa cells incubated with HEK293T-CD63-GFP TSU and PSU sEVs (F1–F5) containing EV-DNA-EdU, the HEK293T-CD63-GFP genomic DNA-EdU (negative control), and imaged using confocal microscopy as illustrated in Figure 3A. As expected, no signal was found on cells incubated with genomic DNA-EdU (data not shown). Comparatively, we found that TSU sEV fractions F2 and F3 could deliver DNA efficiently in HeLa recipient cells both in the cytoplasm and in the nucleus. However, it is negligible in the F1 TSU fraction that contains fewer sEVs and on all PSU sEV fractions (F1–F5) (Figure 3B). Unlike before, we did not observe much GFP fluorescence along with the EdU signal. This could be because the copper ions present in the click-it EdU staining kit might quench the GFP fluorescence, as pointed out by the manufacturer (Figure 3C). In addition, we confirmed that the isolated sEVs are devoid of free EdU since the same effect that we observed in the positive staining control (HeLa cells incubated with EdU for 2 h) was not found in sEV-treated HeLa cells (Figure 3D). To evaluate the efficiency of EV-DNA transfer, we quantified the level of EdU (red fluorescence) derived from various TSU and PSU EV-DNA on HeLa recipient cells, and the data are outlined in Figure 3E. It is apparent that F2 and F3 TSU sEVs are more efficient in delivering DNA into the recipient cells than other fractions. 

To precisely visualize and measure the amount of EV-DNA localized in various cell compartments such as the cell membrane, cytoplasm, and nucleus, we analyzed the reconstructed confocal z-stacks of confocal images containing EV-DNA first-ever using Imaris software. Briefly, deconvolution of data sets was performed using Huygens, and the Imaris surface function was used to distinguish different cell compartments, which helped quantify accurately the amount of EV-DNA localized inside the recipient cells, avoiding false-positive results or artifacts generated from 2D microscopy (Figure 3F). Indeed, we observed that only ~50% of EV-DNA from F2 and F3 and 25% from F4 successfully passed through the cell membrane barrier and localized inside the recipient cells, while others were found outside the cells and at the cell membrane. We found nearly the same amount of EV-DNA (F2 and F3) inside the recipient cells both in the cytoplasm and in the nucleus. However, it is significantly less in F4, which explains that there is a different DNA population present in this fraction (Figure 3G). To confirm if the EV-DNA-EdU transfer approach could be used for any disease model, we incubated leukemia cell line MV4-11-derived sEVs containing EV-DNA-EdU with mouse mesenchymal stromal cells, OP9. Indeed, we observed that MV4-11-EV-DNA was transferred into OP9 cells, which shows that this approach could be used to evaluate the EV-DNA role in cell–cell communication in leukemia (Figure 3H). Next, to analyze EV-DNA organization at a single vesicle level, we imaged HEK-CD63-GFP TSU sEVs (F2 and F3) mounted on poly-L lysine coated coverslips using super-resolution microscopy (Figure 3I). Indeed, we observed that most of the EV-DNA (red) signal was found next to CD63+ EVs (green), indicating that these are intravesicular EV-DNA fragments protected inside the vesicles. However, a small proportion of EV-DNA co-localized with CD63 GFP + EVs revealing the possibility that they are extra vesicular DNA.

### 3.4. Characterization of TSU EV-DNA 

**Figure 4:**
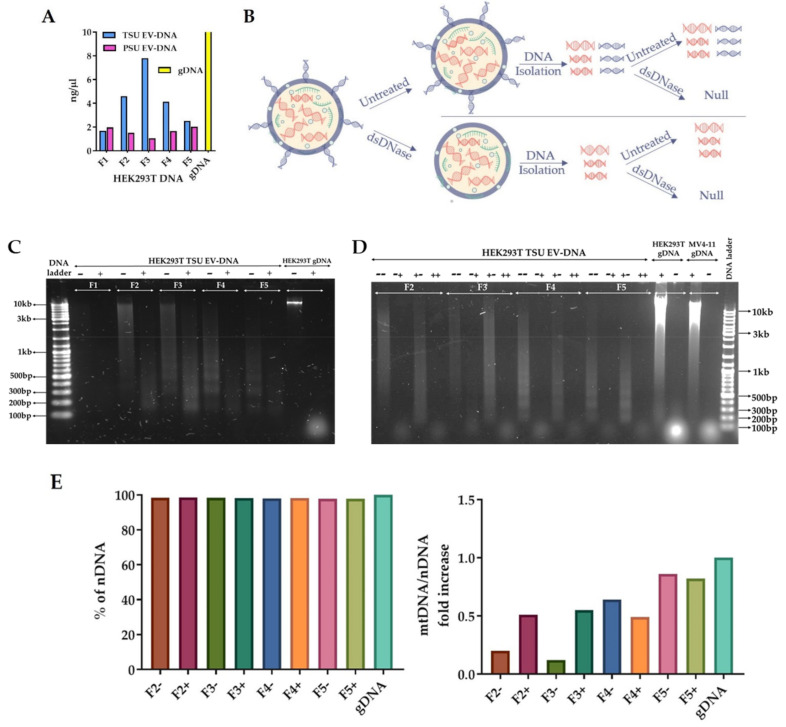
Detailed characterization of EV-DNA. (**A**) Comparison of HEK293T TSU and PSU EV-DNA concentration. (**B**) Scheme displaying the steps involved in the analysis of extravesicular and intravesicular EV-DNA using single and double dsDNAse digestion. (**C**) EV-DNA derived from HEK293T TSU sEVs (F1–F5) pre-treated with and without dsDNAse to demonstrate extravesicular EV-DNA fragments. (**D**) Further treatment of dsDNAse to reveal vesicle protected dsDNA. (**E**) Next-generation sequencing (NGS) analysis of HEK293T-EV-DNA. Percentage of nuclear or genomic DNA and the ratio of host mitochondrial to nuclear DNA fold increase were determined.

Knowing that TSU EV-DNA is actively taken up by the recipient cells, we included only TSU sEVs for further downstream analyses. We analyzed the DNA of TSU sEVs (HEK293T) (F1–F5) more elaborately to characterize vesicle protected and exposed non-vesicular DNA either free or sticking on the surface of the EVs using dsDNase digestion as previously published [5] (Figure 4A,B). EV-DNA samples were stained with SYBR green and loaded onto an agarose gel (Figure 4C). We observed in F2 that high molecular weight DNA fragments larger than 2–10 kb were digested after dsDNase exposure that specially digests only dsDNA. However, in F3, some large DNA fragments (2–10 kb) were protected from dsDNase. Conversely, in F4 and F5, we found an apparent decrease in the level of EV-DNA after dsDNase digestion, indicating that mostly extra vesicular EV-DNA is present in these fractions. In addition, the ideal size of circulating or cell-free DNA fragments ranging between 120–220 bp was observed only in non-sEV fractions (F4 and F5) but not in sEV fractions (F2 and F3) before dsDNase treatment [42,43]. Altogether, it suggests that sEV fractions, F2 and F3, are devoid of cell-free DNA, and primarily large DNA fragments 2–10 kb exist on the outer surface of sEVs, and 220 bp- 2 kb DNA fragments are encapsulated inside sEVs. 

To further investigate if the intravesicular EV-DNA fragments from various HEK293T TSU sEV fractions are double stranded, we digested them with dsDNase again and analyzed them in the same manner as before (Figure 4D). In line with our previous findings [5], we found that intravesicular EV-DNA present in HEK293T TSU sEV fractions (F2–F5) is completely double stranded. To know the details of vesicle and non-vesicle protected DNA, HEK293T EV-DNA samples pre-treated with and without dsDNase were sequenced using NGS platform. Confirming the previous reports, we observed that both vesicle and non-vesicle-protected EV-DNA fragments are mainly derived from the host cell genome; however, a small proportion is derived from the mitochondria (Figure 4E) [27,44,45,46]. Furthermore, we observed that there is an increase in the level of mtDNA on the EV-DNA samples pre-treated with dsDNase, which is in accordance with the finding observed by Sansone et al., 2017 [47].

### 3.5. Association of EV-DNA with Cytoplasmic DNA Sensors and Endosomal Proteins

**Figure 5:**
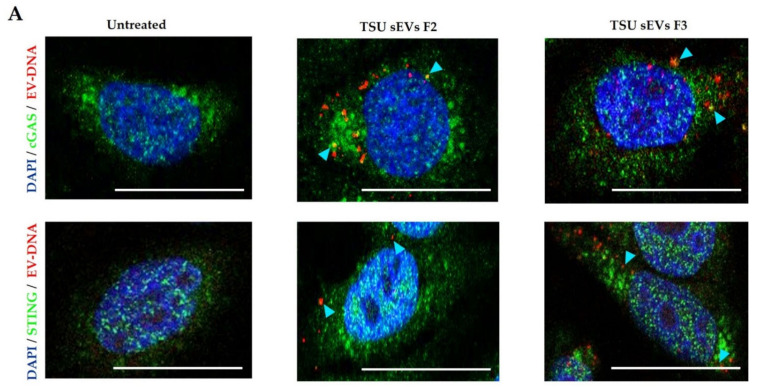

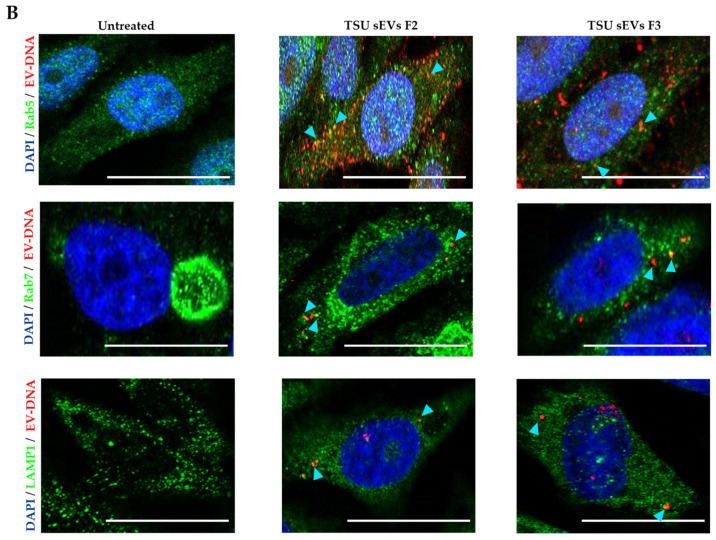
Association of EV-DNA with various cytoplasmic proteins. Staining of HeLa cells with and without HEK293T TSU sEVs (F2 and F3) containing EV-DNA-EdU for (**A**) cGAS and STING (**B**) Rab5, Rab7, and LAMP1. Representative confocal images showing cell nuclei (DAPI- blue), endosomal proteins, and dsDNA sensors (green). Blue arrows show co-localization (yellow) of EV-DNA with various cytoplasmic proteins. Scale bars: 10 µm.

In recent years, it was found that DNA from various sources could enter the cytosol and activate the cGAS-STING pathway: a DNA-driven immune response critical in host defense, inflammation, and tumor immunity [29,30,48]. Interestingly, we observed the co-localization of cGAS and STING with EV-DNA in HeLa cells, suggesting their molecular interaction (Figure 5A).

To determine if EV-DNA is considered to be a potential threat inside the recipient cells directing towards endosomal fusion and degradation in the lysosomal compartment, HeLa cells incubated with EdU-labeled F2 and F3 HEK293T TSU sEVs were stained for Rab5 and Rab7 proteins (early and late endosomal markers) and LAMP1 (lysosomal autophagy marker). Further elucidating the previous observations by Rappa et al. [49], we found that a portion of EV-DNA associates with Rab5 and Rab7 (Figure 5B). In addition, the co-localization of EV-DNA with LAMP-1 highlights that they are in the process of degradation.

## 4. Discussion

Using differential UC and polymer-based EV isolation methods, many studies have demonstrated EV-DNA as a potential biomarker for various diseases [5,7,21,50,51] and the functional role of EV-DNA in cell–cell communication [30,47,52]. Although these EV isolation methods provide good EV yield to perform diagnostic studies, we claim that these isolated EVs are not suitable to perform EV-DNA-based functional studies since these methods are known to co-isolate cell-free DNA (cfDNA) and apoptotic bodies [23,24,25,37,53]. Because of these obstacles, one cannot illustrate exactly if the observed physiological function in the recipient cells is due to EV-DNA only, not by any other contaminants [3]. To address this problem, we applied a simple benchtop method to enrich sEVs from non-EV particles using a state-of-the-art combination of tangential flow filtration, size-exclusion chromatography, and ultrafiltration (TSU). Out of five TSU sEVs fractions (F1–F5), fractions (F2 and F3) fulfill the EV criteria recommended by MISEV2018 [3]. Further, TSU F2 and F3 provide sEVs qualified to perform EV-DNA functional studies.

To reveal the distribution of EV-DNA in the recipient cells, we employed the 3D reconstruction of confocal z-stacks using Imaris software. Contrary to 2D imaging data, we found through 3D analysis that almost the same amount of EV-DNA from F2 and F3 entered the cytoplasm and the nucleus of recipient cells (Figure 3E,G). In addition, from this analysis, we revealed something unique: the recipient cell membrane barrier prevents almost 50 percent of F3-EV-DNA from getting inside. There may be other cofactors deciding the entry of different EV-DNA fragments into the recipient cells, which requires further investigation. Collectively, 3D image analysis using Imaris helps EV researchers in the future to avoid reporting false-positive results generated from confocal microscopy images taken in a single plane of focus [54]. Next, we utilized CD63+ EVs and EV-DNA-EdU to study the arrangement of EVs and EV-DNA at a single vesicle level employing super-resolution microscopy. As expected, we revealed that not all EV-DNA was associated with the EV surface marker, CD63, since EV-DNA was mainly localized inside the vesicles (Figure 3I). 

Lázaro-Ibáñez et al., 2019 isolated sEVs using UC-based iodixanol density separation; however, it is unclear whether radiocontrast agents such as iodixanol used for separation will be entirely removed from EV preparation [27]. In our case, we observed that the PEG used for the precipitation of sEVs influences the EV uptake and EV-DNA entry into the recipient cells. In addition, they showed that DNA isolated from both low-density and high-density fractions possessed nucleosomal patterns [27]. On the other hand, Vagner et al., 2018 showed that large EVs isolated from prostate cancer cells are enriched in chromosomal DNA, suggesting that most genomic DNA detected in large EVs is nucleosomal, most probably originating in the extracellular space from apoptotic or necrotic cells [55]. Supporting these facts only to a limited extent, we found that isolated small EVs are free from apoptotic bodies; however, the cell-free DNA population with a nucleosomal pattern was observed only in fractions, F4 and F5, which contain fewer small EVs (Figure 4C). Besides, NGS analysis confirmed that mitochondria DNA fragments are enriched more in non-EV fraction F5. Taken together, these observations hint that mostly EV-DNA present in F2 and F3 are genomic DNA fragments packaged inside sEVs, not as a circulating cell-free DNA derived from dead cells or cancer cells. 

We know that viral dsDNA activates some pathways during active virus infection, hijacking the host defense machinery to support their survival in the human body [56,57]. It is not explored whether EV-DNA communicates in the same way in our human body to drive cancer progression, metastasis, and cancer relapse. Bakhoum et al., 2018 revealed for the first time that there is an unexpected link between chromosome instability and the release of free DNA in the cytoplasm for the activation of cytosolic DNA sensing pathways and metastasis [29]. Furthermore, only one study has addressed so far that EV-DNA is involved in cell–cell communication between T cells and dendritic cells. In this study, they used UC to isolate EVs that can be contaminated with other non-EV populations. They showed that activation of the cGAS/STING/IRF3 signaling axis in priming the dendritic cells is an exclusive function of EV-DNA released by T-cells [30]. Similarly, we observed that EV-DNA associates with the cytoplasmic sensors cGAS and STING. However, in the future, after obtaining sEVs using TSU, we need to evaluate if EV-DNA association with cGAS-STING plays an essential role in cancer. In addition, whether DNA associated with F2 and F3 is linked with histones needs to be determined. It could be possible that histones associated with EV-DNA and other cofactors collaborated with EV-DNA might be essential for EV-DNA function in the recipient cells, driving towards cancer progression or metastasis.

## 5. Conclusions

TSU serves as a better choice for EV isolation when EV-DNA functional studies need to be executed since it provides a small EV preparation deficient in cell-free DNA and apoptotic bodies, unlike other commonly used methods. In addition, the TSU-EV-DNA-EdU labeling approach, along with three-dimensional microscope image analysis using Imaris, helps EV researchers to evaluate the EV-DNA role in cell–cell communication in cancer. 

## Data Availability

All authors declare that the data presented in this study are available within the article and its supplementary information files or upon reasonable requests to the corresponding author.

## Supplementary Materials

The following supporting information can be downloaded at https://www.mdpi.com/article/10.3390/cancers14092068/s1, Figure S1: A: HeLa sEVs NTA and micro BCA data; B: HeLa TSU and PSU sEVs purity; C: HeLa sEVs TEM images; D: HeLa sEVs average diameter; E: Original western blot images; F: Single beads selection; G: CD9+ population selection; H: Purity based on EV tetraspanins and micro BCA value; I: HeLa EV-DNA concentration.

## Abbreviations

EVs	Extracellular vesicles
sEVs	Small extracellular vesicles
dsDNA	Double-stranded DNA
EV-DNA	Small extracellular vesicles DNA
cfDNA	Cell-free DNA
TSU	Tangential flow filtration + Size -exclusion chromatography + Ultrafiltration
PSU	Polyethylene glycol precipitation + Size-exclusion chromatography + Ultrafiltration
MSCs	Mesenchymal stromal cells
BM	Bone marrow
cGAS	Cyclic GMP-AMP synthase
STING	Stimulator of interferon genes
UC	Ultracentrifugation
SEC	Size-exclusion chromatography
UF	Ultrafiltration
TFF	Tangential flow filtration
PEG	Polyethylene glycol
IRF3	Interferon regulatory factor 3
AML	Acute myeloid leukemia
GFP	Green fluorescent protein
FBS	Fetal bovine serum
CCM	Cell conditioned media
NTA	Nanoparticle tracking analysis
TEM	Transmission electron microscopy
RT	Room temperature
MFI	Mean fluorescence intensity
FLT3	Fms-like tyrosine kinase 3
NPM1	Nucleophosmin 1
EdU	5-ethynyl-2′-deoxyuridine
WGA	Wheat germ agglutinin
NGS	Next-generation sequencing
SEM	Standard error mean
LAMP1	Lysosomal-associated membrane protein 1
Rab5/7	Ras-related proteins

## References

[1] Ghanam J., Chetty V.K., Barthel L., Reinhardt D., Hoyer P.F., Thakur B.K. (2022). DNA in extracellular vesicles: From evolution to its current application in health and disease. Cell Biosci..

[2] Elzanowska J., Semira C., Costa-Silva B. (2021). DNA in extracellular vesicles: Biological and clinical aspects. Mol. Oncol..

[3] Thery C., Witwer K.W., Aikawa E., Alcaraz M.J., Anderson J.D., Andriantsitohaina R., Antoniou A., Arab T., Archer F., Atkin-Smith G.K. (2018). Minimal information for studies of extracellular vesicles 2018 (MISEV2018): A position statement of the International Society for Extracellular Vesicles and update of the MISEV2014 guidelines. J. Extracell. Vesicles.

[4] Thery C., Amigorena S., Raposo G., Clayton A. (2006). Isolation and characterization of exosomes from cell culture supernatants and biological fluids. Curr. Protoc. Cell Biol..

[5] Thakur B.K., Zhang H., Becker A., Matei I., Huang Y., Costa-Silva B., Zheng Y., Hoshino A., Brazier H., Xiang J. (2014). Double-stranded DNA in exosomes: A novel biomarker in cancer detection. Cell Res..

[6] Valadi H., Ekstrom K., Bossios A., Sjostrand M., Lee J.J., Lotvall J.O. (2007). Exosome-mediated transfer of mRNAs and microRNAs is a novel mechanism of genetic exchange between cells. Nat. Cell Biol..

[7] Kahlert C., Melo S.A., Protopopov A., Tang J., Seth S., Koch M., Zhang J., Weitz J., Chin L., Futreal A. (2014). Identification of double-stranded genomic DNA spanning all chromosomes with mutated KRAS and p53 DNA in the serum exosomes of patients with pancreatic cancer. J. Biol. Chem..

[8] Hoshino A., Costa-Silva B., Shen T.L., Rodrigues G., Hashimoto A., Tesic Mark M., Molina H., Kohsaka S., Di Giannatale A., Ceder S. (2015). Tumour exosome integrins determine organotropic metastasis. Nature.

[9] Gao Y., Qin Y., Wan C., Sun Y., Meng J., Huang J., Hu Y., Jin H., Yang K. (2021). Small Extracellular Vesicles: A Novel Avenue for Cancer Management. Front. Oncol..

[10] Droste M., Thakur B.K., Eliceiri B.P. (2020). Tumor-Derived Extracellular Vesicles and the Immune System-Lessons From Immune-Competent Mouse-Tumor Models. Front. Immunol..

[11] Yanez-Mo M., Siljander P.R., Andreu Z., Zavec A.B., Borras F.E., Buzas E.I., Buzas K., Casal E., Cappello F., Carvalho J. (2015). Biological properties of extracellular vesicles and their physiological functions. J. Extracell. Vesicles.

[12] Admyre C., Johansson S.M., Qazi K.R., Filen J.J., Lahesmaa R., Norman M., Neve E.P., Scheynius A., Gabrielsson S. (2007). Exosomes with immune modulatory features are present in human breast milk. J. Immunol..

[13] Malkin E.Z., Bratman S.V. (2020). Bioactive DNA from extracellular vesicles and particles. Cell Death Dis..

[14] Pitt J.M., Kroemer G., Zitvogel L. (2016). Extracellular vesicles: Masters of intercellular communication and potential clinical interventions. J. Clin. Investig..

[15] Maas S.L.N., Breakefield X.O., Weaver A.M. (2017). Extracellular Vesicles: Unique Intercellular Delivery Vehicles. Trends Cell Biol..

[16] Liu C., Yu S., Zinn K., Wang J., Zhang L., Jia Y., Kappes J.C., Barnes S., Kimberly R.P., Grizzle W.E. (2006). Murine mammary carcinoma exosomes promote tumor growth by suppression of NK cell function. J. Immunol..

[17] Umezu T., Tadokoro H., Azuma K., Yoshizawa S., Ohyashiki K., Ohyashiki J.H. (2014). Exosomal miR-135b shed from hypoxic multiple myeloma cells enhances angiogenesis by targeting factor-inhibiting HIF-1. Blood.

[18] Moore M., Davachi F., Bongo L., Seruvugo H., Mushiya K., Roy J.A., Mambu ma D. (1989). New parameters for evaluating oral rehydration therapy: One year’s experience in a major urban hospital in Zaire. J. Trop. Pediatr..

[19] Hur J.Y., Kim H.J., Lee J.S., Choi C.M., Lee J.C., Jung M.K., Pack C.G., Lee K.Y. (2018). Extracellular vesicle-derived DNA for performing EGFR genotyping of NSCLC patients. Mol. Cancer.

[20] Klump J., Phillipp U., Follo M., Eremin A., Lehmann H., Nestel S., von Bubnoff N., Nazarenko I. (2018). Extracellular vesicles or free circulating DNA: Where to search for BRAF and cKIT mutations?. Nanomedicine.

[21] Kontopoulou E., Strachan S., Reinhardt K., Kunz F., Walter C., Walkenfort B., Jastrow H., Hasenberg M., Giebel B., von Neuhoff N. (2020). Evaluation of dsDNA from extracellular vesicles (EVs) in pediatric AML diagnostics. Ann. Hematol..

[22] Maire C.L., Fuh M.M., Kaulich K., Fita K.D., Stevic I., Heiland D.H., Welsh J.A., Jones J.C., Gorgens A., Ricklefs T. (2021). Genome-wide methylation profiling of glioblastoma cell-derived extracellular vesicle DNA allows tumor classification. Neuro-Oncology.

[23] Sidhom K., Obi P.O., Saleem A. (2020). A Review of Exosomal Isolation Methods: Is Size Exclusion Chromatography the Best Option?. Int. J. Mol. Sci..

[24] Liangsupree T., Multia E., Riekkola M.L. (2021). Modern isolation and separation techniques for extracellular vesicles. J. Chromatogr. A.

[25] Furi I., Momen-Heravi F., Szabo G. (2017). Extracellular vesicle isolation: Present and future. Ann. Transl. Med..

[26] Lobb R.J., Becker M., Wen S.W., Wong C.S., Wiegmans A.P., Leimgruber A., Moller A. (2015). Optimized exosome isolation protocol for cell culture supernatant and human plasma. J. Extracell. Vesicles.

[27] Lazaro-Ibanez E., Lasser C., Shelke G.V., Crescitelli R., Jang S.C., Cvjetkovic A., Garcia-Rodriguez A., Lotvall J. (2019). DNA analysis of low- and high-density fractions defines heterogeneous subpopulations of small extracellular vesicles based on their DNA cargo and topology. J. Extracell. Vesicles.

[28] Tian T., Zhu Y.L., Zhou Y.Y., Liang G.F., Wang Y.Y., Hu F.H., Xiao Z.D. (2014). Exosome uptake through clathrin-mediated endocytosis and macropinocytosis and mediating miR-21 delivery. J. Biol. Chem..

[29] Bakhoum S.F., Ngo B., Laughney A.M., Cavallo J.A., Murphy C.J., Ly P., Shah P., Sriram R.K., Watkins T.B.K., Taunk N.K. (2018). Chromosomal instability drives metastasis through a cytosolic DNA response. Nature.

[30] Torralba D., Baixauli F., Villarroya-Beltri C., Fernandez-Delgado I., Latorre-Pellicer A., Acin-Perez R., Martin-Cofreces N.B., Jaso-Tamame A.L., Iborra S., Jorge I. (2018). Priming of dendritic cells by DNA-containing extracellular vesicles from activated T cells through antigen-driven contacts. Nat. Commun..

[31] Ludwig A.K., De Miroschedji K., Doeppner T.R., Borger V., Ruesing J., Rebmann V., Durst S., Jansen S., Bremer M., Behrmann E. (2018). Precipitation with polyethylene glycol followed by washing and pelleting by ultracentrifugation enriches extracellular vesicles from tissue culture supernatants in small and large scales. J. Extracell. Vesicles.

[32] Chen S.Y., Bestvater F., Schaufler W., Heintzmann R., Cremer C. (2018). Patterned illumination single molecule localization microscopy (piSMLM): User defined blinking regions of interest. Opt. Express.

[33] Ovesny M., Krizek P., Borkovec J., Svindrych Z., Hagen G.M. (2014). ThunderSTORM: A comprehensive ImageJ. plug-in for PALM and STORM data analysis and super-resolution imaging. Bioinformatics.

[34] Schindelin J., Arganda-Carreras I., Frise E., Kaynig V., Longair M., Pietzsch T., Preibisch S., Rueden C., Saalfeld S., Schmid B. (2012). Fiji: An open-source platform for biological-image analysis. Nat. Methods.

[35] Bolger A.M., Lohse M., Usadel B. (2014). Trimmomatic: A flexible trimmer for Illumina sequence data. Bioinformatics.

[36] Li H. (2011). A statistical framework for SNP calling, mutation discovery, association mapping and population genetical parameter estimation from sequencing data. Bioinformatics.

[37] Webber J., Clayton A. (2013). How pure are your vesicles?. J. Extracell. Vesicles.

[38] Kornilov R., Puhka M., Mannerstrom B., Hiidenmaa H., Peltoniemi H., Siljander P., Seppanen-Kaijansinkko R., Kaur S. (2018). Efficient ultrafiltration-based protocol to deplete extracellular vesicles from fetal bovine serum. J. Extracell. Vesicles.

[39] Suarez H., Gamez-Valero A., Reyes R., Lopez-Martin S., Rodriguez M.J., Carrascosa J.L., Cabanas C., Borras F.E., Yanez-Mo M. (2017). A bead-assisted flow cytometry method for the semi-quantitative analysis of Extracellular Vesicles. Sci. Rep..

[40] Midekessa G., Godakumara K., Ord J., Viil J., Lattekivi F., Dissanayake K., Kopanchuk S., Rinken A., Andronowska A., Bhattacharjee S. (2020). Zeta Potential of Extracellular Vesicles: Toward Understanding the Attributes that Determine Colloidal Stability. ACS Omega.

[41] Frohlich E. (2012). The role of surface charge in cellular uptake and cytotoxicity of medical nanoparticles. Int. J. Nanomed..

[42] Chen E., Cario C.L., Leong L., Lopez K., Marquez C.P., Chu C., Li P.S., Oropeza E., Tenggara I., Cowan J. (2021). Cell-free DNA concentration and fragment size as a biomarker for prostate cancer. Sci. Rep..

[43] Alcaide M., Cheung M., Hillman J., Rassekh S.R., Deyell R.J., Batist G., Karsan A., Wyatt A.W., Johnson N., Scott D.W. (2020). Evaluating the quantity, quality and size distribution of cell-free DNA by multiplex droplet digital PCR. Sci. Rep..

[44] Soltesz B., Urbancsek R., Pos O., Hajas O., Forgacs I.N., Szilagyi E., Nagy-Balo E., Szemes T., Csanadi Z., Nagy B. (2019). Quantification of peripheral whole blood, cell-free plasma and exosome encapsulated mitochondrial DNA copy numbers in patients with atrial fibrillation. J. Biotechnol..

[45] Keseru J.S., Soltesz B., Lukacs J., Marton E., Szilagyi-Bonizs M., Penyige A., Poka R., Nagy B. (2019). Detection of cell-free, exosomal and whole blood mitochondrial DNA copy number in plasma or whole blood of patients with serous epithelial ovarian cancer. J. Biotechnol..

[46] Guescini M., Genedani S., Stocchi V., Agnati L.F. (2010). Astrocytes and Glioblastoma cells release exosomes carrying mtDNA. J. Neural Transm..

[47] Sansone P., Savini C., Kurelac I., Chang Q., Amato L.B., Strillacci A., Stepanova A., Iommarini L., Mastroleo C., Daly L. (2017). Packaging and transfer of mitochondrial DNA via exosomes regulate escape from dormancy in hormonal therapy-resistant breast cancer. Proc. Natl. Acad. Sci. USA.

[48] Motwani M., Pesiridis S., Fitzgerald K.A. (2019). DNA sensing by the cGAS-STING pathway in health and disease. Nat. Rev. Genet..

[49] Rappa G., Santos M.F., Green T.M., Karbanova J., Hassler J., Bai Y., Barsky S.H., Corbeil D., Lorico A. (2017). Nuclear transport of cancer extracellular vesicle-derived biomaterials through nuclear envelope invagination-associated late endosomes. Oncotarget.

[50] Cambier L., Stachelek K., Triska M., Jubran R., Huang M., Li W., Zhang J., Li J., Cobrinik D. (2021). Extracellular vesicle-associated repetitive element DNAs as candidate osteosarcoma biomarkers. Sci. Rep..

[51] Vaidya M., Bacchus M., Sugaya K. (2018). Differential sequences of exosomal NANOG DNA as a potential diagnostic cancer marker. PLoS ONE.

[52] Cai J., Han Y., Ren H., Chen C., He D., Zhou L., Eisner G.M., Asico L.D., Jose P.A., Zeng C. (2013). Extracellular vesicle-mediated transfer of donor genomic DNA to recipient cells is a novel mechanism for genetic influence between cells. J. Mol. Cell Biol..

[53] Gamez-Valero A., Monguio-Tortajada M., Carreras-Planella L., Franquesa M., Beyer K., Borras F.E. (2016). Size-Exclusion Chromatography-based isolation minimally alters Extracellular Vesicles’ characteristics compared to precipitating agents. Sci. Rep..

[54] Gautier M.K., Ginsberg S.D. (2021). A method for quantification of vesicular compartments within cells using 3D reconstructed confocal z-stacks: Comparison of ImageJ. and Imaris to count early endosomes within basal forebrain cholinergic neurons. J. Neurosci. Methods.

[55] Vagner T., Spinelli C., Minciacchi V.R., Balaj L., Zandian M., Conley A., Zijlstra A., Freeman M.R., Demichelis F., De S. (2018). Large extracellular vesicles carry most of the tumour DNA circulating in prostate cancer patient plasma. J. Extracell. Vesicles.

[56] Shlomai A., Schwartz R.E., Ramanan V., Bhatta A., de Jong Y.P., Bhatia S.N., Rice C.M. (2014). Modeling host interactions with hepatitis B virus using primary and induced pluripotent stem cell-derived hepatocellular systems. Proc. Natl. Acad. Sci. USA.

[57] Morissette G., Flamand L. (2010). Herpesviruses and chromosomal integration. J. Virol..

