# Supplementary material for: Efficient Small Extracellular Vesicles (EV) Isolation Method and Evaluation of EV-Associated DNA Role in Cell–Cell Communication in Cancer
Source: Cancers (Basel). 2022 Apr 20;14(9):2068. doi: 10.3390/cancers14092068 (PMC9099953; doi:10.3390/cancers14092068)

Supplementary Figure S1

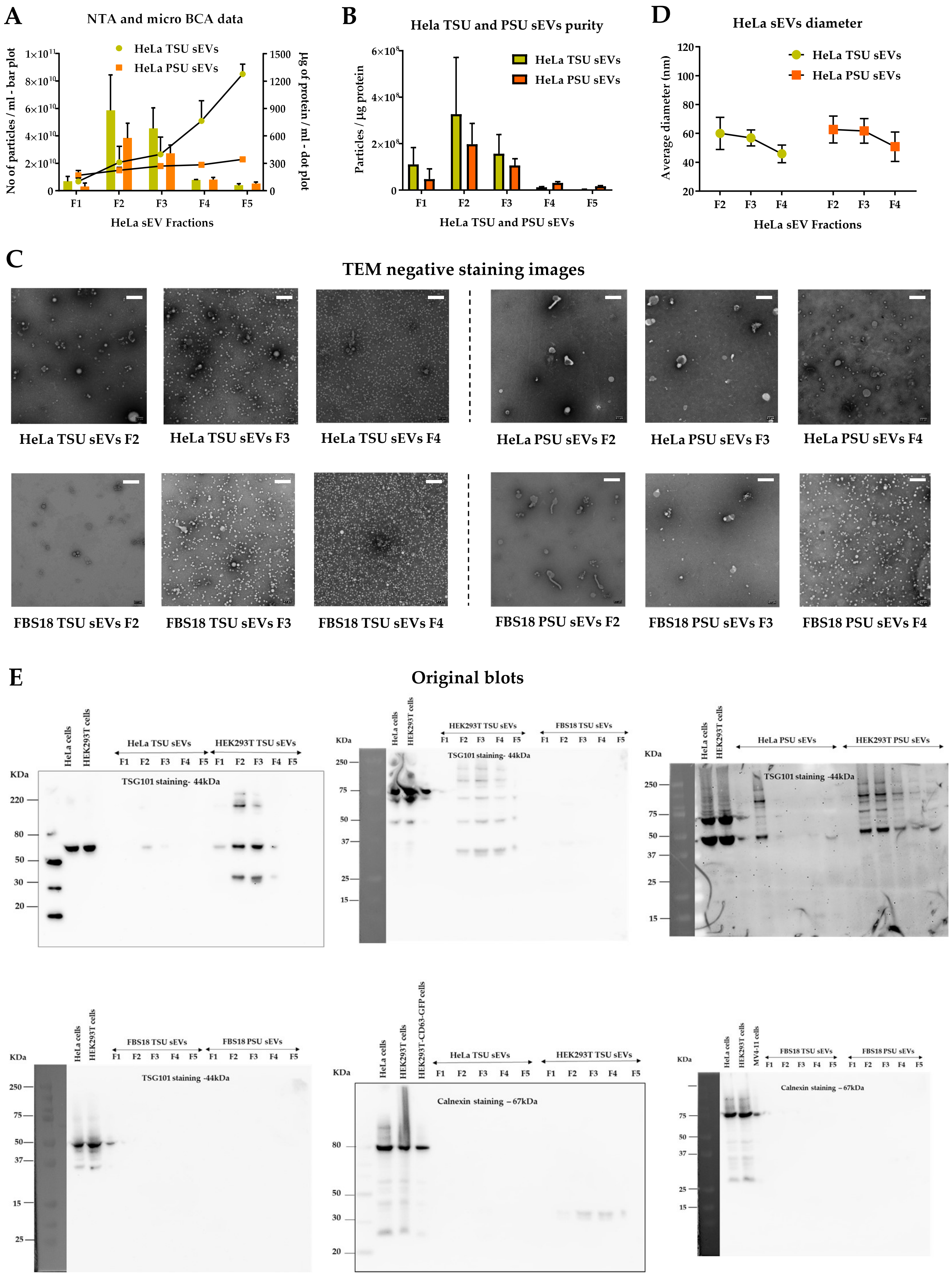

Supp. Figure S1 (contd.)

E

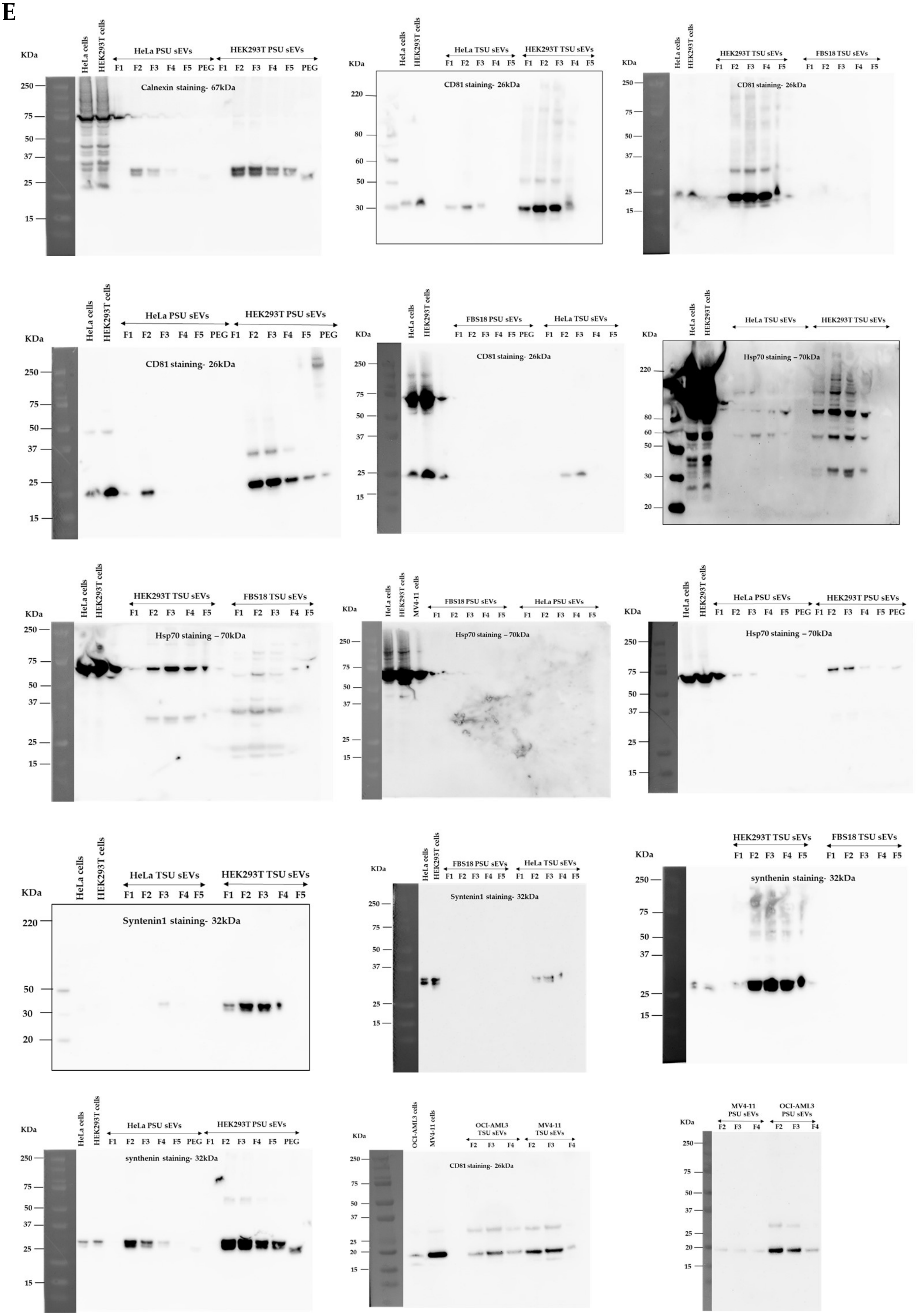

Supp. Figure S1 (contd.)

E

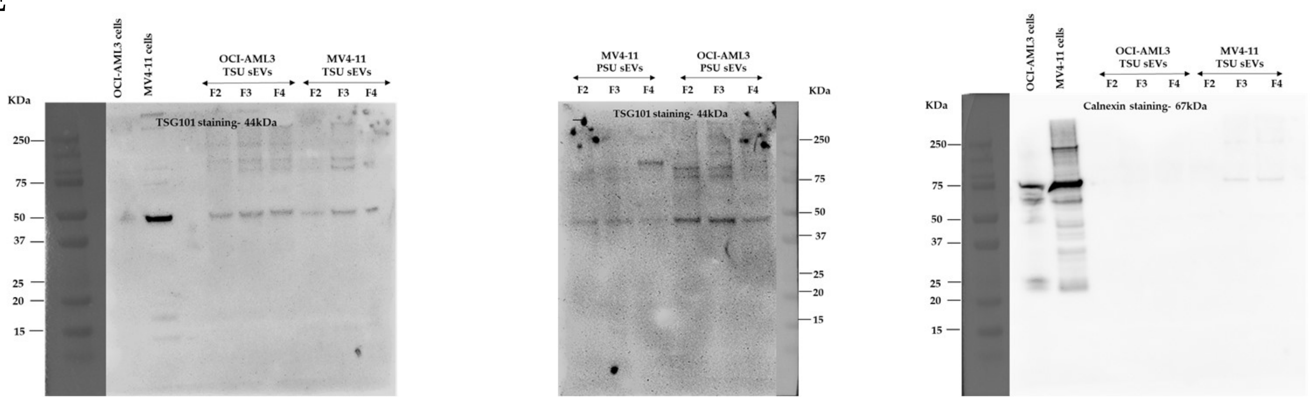

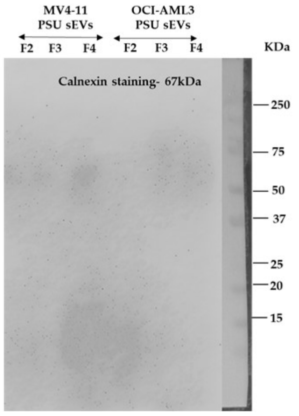

F Selection of single beads

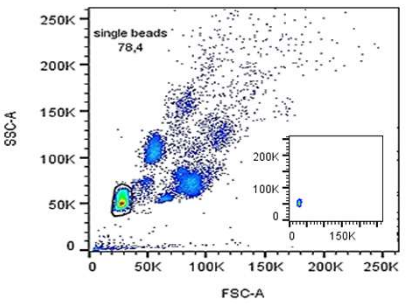

G Selection of CD9+ population

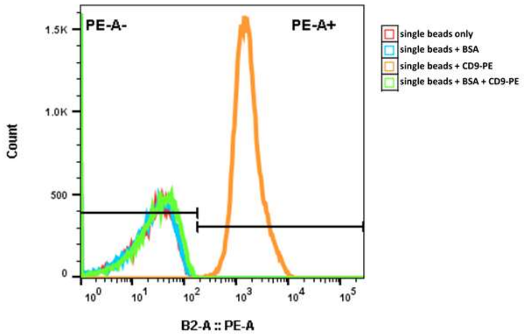

H Purity based on EV tetraspanins and micro-BCA value

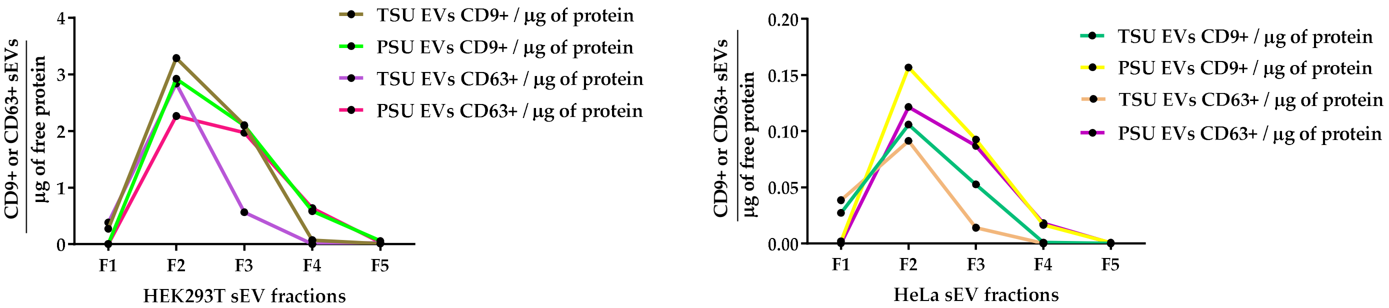

I DNA concentration

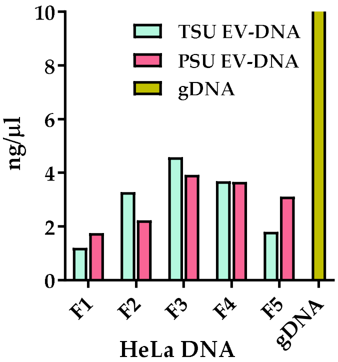

Supplement: Supplementary file 1 [file cancers-14-02068-s001.zip › cancers-1673264-supplementary.pdf]
